# Social and occupational recovery in early psychosis: a systematic review and meta-analysis of psychosocial interventions

**DOI:** 10.1017/S003329172100341X

**Published:** 2023-04

**Authors:** E. Frawley, M. Cowman, M. Lepage, G. Donohoe

**Affiliations:** 1 Centre for Neuroimaging, Cognition & Genomics (NICOG), School of Psychology, National University of Ireland Galway, Galway, Ireland; 2 Prevention and Early Intervention Program for Psychosis, Douglas Mental Health University Institute, Montreal, Canada; 3 Department of Psychiatry, McGill University, Montreal, Canada

**Keywords:** Early psychosis, occupational function, psychosocial intervention, recovery, social function

## Abstract

**Background:**

Psychosis, even in its early stages, ranks highly among the causes of disability worldwide, resulting in an increased focus on improved recovery of social and occupational functioning. This study aimed to provide an estimate of the effectiveness of psychosocial interventions for improving functioning in early psychosis. We also sought evidence of superiority between intervention approaches.

**Methods:**

An electronic search was conducted using PubMed and PsycINFO to identify original articles reporting on trials of psychosocial interventions in early-stage psychosis, published up to December 2020 and is reported following PRISMA guidelines. Data were extracted on validated measures of functioning from included studies and pooled standardised mean difference (SMD) was estimated.

**Results:**

In total, 31 studies involving 2811 participants were included, focusing on: cognitive behavioural therapy for psychosis (CBTp), family-based therapy, supported employment, cognitive remediation training (CRT) and multi-component psychosocial interventions. Across interventions, improved function was observed (SMD = 0.239; 95% confidence interval 0.115–0.364, *p* < 0.001). Effect sizes varied by intervention type, stage of illness, length and duration of treatment and outcome measure used. In particular, interventions based on CRT significantly outperformed symptom-focused CBT interventions, while multi-component interventions were associated with largest gains.

**Conclusions:**

Psychosocial interventions, particularly when provided as part of a multi-component intervention model and delivered in community-based settings are associated with significant improvements in social and occupational function. This review underscores the value of sensitively tracking and targeting psychosocial function as part of the standard provided by early intervention services.

## Introduction

Psychosis, even in its early stages, is associated with significant disability, causing it to be ranked ahead of paraplegia and blindness in those aged 18–35 in terms of years lived with disability. Current pharmacological treatments target positive symptoms (hallucinations and delusions) of psychosis, but not other features of illness, including negative and affective symptoms and cognitive deficits, which more accurately predict functional outcome than positive symptoms alone (Green, 2016). Consequently, even after successful treatment of positive symptoms, little benefit to functional outcome may result, suggesting a need to expand the range of treatment targets (Hodgekins et al., 2015; Malla & Mcgorry, 2019).

Despite this, psychosocial interventions for psychosis have often focused only on clinical/symptom improvement as the main outcome, leading to a conclusion of equivalence between psychosocial treatments in terms of modest treatment benefits (Fusar-Poli et al., 2015). However, focusing on only one illness dimension (e.g. positive symptom severity), ignores the range of factors contributing to overall loss of social/occupational function, measured in terms of reduced social engagement and significant underemployment (~20% of individuals with psychosis go on to independent employment). In first-episode psychosis, a meta-analysis by Santesteban-Echarri et al. (2017) found that duration of untreated psychosis, cognitive function and remission of positive and negative symptoms were each *independently* related to functional recovery (Santesteban-Echarri et al., 2017). Similarly, Stouten, Veling, Laan, Van Der Helm, and Van Der Gaag (2017) found that poorer functioning was associated with higher levels of negative symptoms, poorer cognitive function and poorer social cognition (explaining 39.4% of variance) (Stouten et al., 2017). We observed similar results in first-episode psychosis, and also identified premorbid adjustment as another relevant factor (Jordan et al., 2014, 2018). By contrast, affective or positive symptoms did not have a marked impact on psychosocial functioning.

Here, we present a systematic review and meta-analysis of psychosocial interventions delivered during the early phase of psychosis, i.e. either during the high-risk stage, or within the 5 years after first diagnosis based on a range of outcomes relevant to social and occupational disability and recovery. We sought to include studies which *evaluated changes in level of social and occupational function* in early psychosis, either directly by targeting some aspect of function, or indirectly by targeting clinical or contextual factors negatively impacting on function. These factors included: (1) clinical symptom severity, (2) hospital readmission rates, (3) levels of distress, (4) quality of life, (5) level of cognitive function and (6) level of social and occupational function. In addition to reviewing evidence for the efficacy and/or effectiveness of these interventions, we also sought evidence of superiority between these approaches while taking into consideration whether social and occupational functioning was considered a primary or secondary outcome in included studies.

## Method

### Study selection

An electronic search was conducted using PubMed and PsycINFO to identify original articles reporting on trials of psychosocial interventions in early-stage psychosis, published up to December 2020. Early-stage psychosis was defined as including the high-risk stage, and anytime within 5 years of a first diagnosis of psychotic disorder. Psychosocial interventions were defined as psychologically and socially orientated interventions which targeted and then evaluated changes in the level of social and occupational function (either as a primary or secondary outcome). Social and occupational functioning was assessed using one or more of the following: (1) global functioning as measured by standardised measures [e.g. global assessment of function (GAF), Social and Occupational Functioning Assessment Scale (SOFAS), Social Functioning Scale (SFS)], Personal Social Performance scale (PSP); and (2) individual definitions of functioning covering one or more of the following areas: vocational functioning, educational functioning, degree of independence and social functioning (i.e. relationships).

### Search strategy

An electronic search was conducted using PubMed and PsycINFO to identify articles investigating the effects of psychosocial interventions on psychosocial function in first-episode psychosis using the following search terms: (‘Early psychosis’ OR ‘clinical high risk’ AND ‘Psychosis’ OR ‘ultra-high risk’ AND ‘Psychosis’) OR (‘first episode psychosis’ OR ‘first episode schizophrenia’ OR ‘recent onset psychosis’ OR ‘recent onset schizophrenia’ OR ‘early psychosis’ OR ‘early schizophrenia’) AND (‘social function*’ OR ‘social outcome*’ OR ‘global function*’ OR ‘global outcome*’ OR ‘community function*’ OR ‘community outcome*’ OR ‘occupational function*’ OR ‘occupational outcome*’ OR ‘work function*’ OR ‘work outcome*’ OR ‘vocational function*’ OR ‘vocational outcome*’ OR ‘recovery’ OR ‘quality of life’ OR ‘employment’ OR ‘global assessment of function’ OR ‘social and occupational functioning assessment scale’ OR ‘functioning scale’ OR ‘disability’) AND (‘psychosocial’ OR ‘psychological’ OR ‘Intervention’ OR ‘therapy’ OR ‘CBT’ or ‘Cognitive behav*’OR ‘CRT’ or ‘Cognitive remed*’ OR ‘ Social’ or ‘Social skills’ OR ‘IPS’ OR ‘Individual placement support’ OR ‘Vocation*’ OR ‘Online’ OR ‘Moderated’ or ‘Moderated support’ OR ‘Family Therapy’ OR ‘Assertive outreach’ OR ‘Outreach’ ‘trial’ OR ‘program’ OR ‘randomised control trial’ OR ‘RCT’ OR ‘pilot’ OR ‘study’).Searches were limited to original, full text articles written in English and published in peer-reviewed journals up to December 2020. The initial electronic search was conducted by two authors (EF and MC). It was fully replicated in a second, independent search. No discrepancies were noted with both search results cross-checked by a third author (GD).

### Quality assurance

The quality assessment of included studies was based on the revised version of the quality evaluation scale employed in our previous reviews as follows: (1) the clinical sample was representative of the target population (eligible cases were recruited in hospitals and/or mental health services settings with a diagnosis based on well-established clinical diagnostic manuals), (2) the clinical sample was appropriately matched to the control group (patients and controls matched for at least two confounding variables: age and/or sex and/or education level, (3) the authors performed sample size calculations and/or power analysis, (4) the study used well-established measures of psychosocial functioning either as a primary or secondary outcome measure, (5) the study provided adequate detail on the psychosocial intervention provided and (6) the authors reported effect sizes and/or confidence intervals (CIs) of their main findings. Each item scored one point if the criterion was met and the overall quality score was a sum of the met criteria (Rokita, Dauvermann, & Donohoe, 2018).

### Data extraction

Data were extracted on validated measures of functioning from included studies. Relevant data extracted also included study and participant characteristics (nature of the intervention, intervention length, follow-up length, control condition, number of sessions, age, percent male, diagnoses, medication use, and illness duration). The authors extracted data independently and discrepancies were resolved by consensus (EF, MC and GD).

### Data analysis

Pooled standardised mean difference (SMD – Cohen's *d*) was estimated with Comprehensive Meta-Analysis Software (CMA), Version 3 (Borenstein, Hedges, Higgins, & Rothstein, 2013). SMD was chosen as the effect size as raw mean and standard deviation scores were provided for most included studies and to allow for the heterogeneity in functional measures used across studies. Due to the variability across studies in length of follow-up assessment, immediately post-intervention data were included in the analyses. For continuous variables, where possible, raw data (pre and post means and standard deviations) was used to estimate effect sizes. Where raw data were unavailable, sample size and *F* statistics were used. Two studies provided dichotomous variables for which events and sample size were used (i.e. employed *v.* unemployed). CMA allows for the inclusion of different data formats in the same analysis (Borenstein, Hedges, Higgins, & Rothstein, 2011). Effect sizes were pooled using random-effects models. Separate analyses were conducted for five different intervention groups, and an overall summary analysis was conducted including all psychosocial intervention studies. For two intervention groups (supported employment and family-based interventions) only three studies were included in the meta-analysis, due to the small number of studies in each group these analyses should be considered exploratory. Subgroup analyses were performed to account for differences in effect size based on participant, intervention and measurement characteristics.

### Heterogeneity and publication bias

Heterogeneity was explored using the *Q* statistic and the *I*^2^ statistics. The *Q* statistic measures the dispersion of all effect sizes about the mean effect size, the *I*^2^ statistic measures the ratio of true variance to total variance (Borenstein et al., 2011). Publication bias was examined by visual inspection of funnel plots, the trim-and-fill method (Duval & Tweedie, 2000) and the regression test (Egger, Smith, Schneider, & Minder, 1997).

### Role of the funding source

The funder of the study had no role in study design, data collection, data analysis, data interpretation or writing of the report. The corresponding author had full access to all the data in the study and had final responsibility for the decision to submit for publication.

## Results

### Study characteristics

**Fig. 1.**
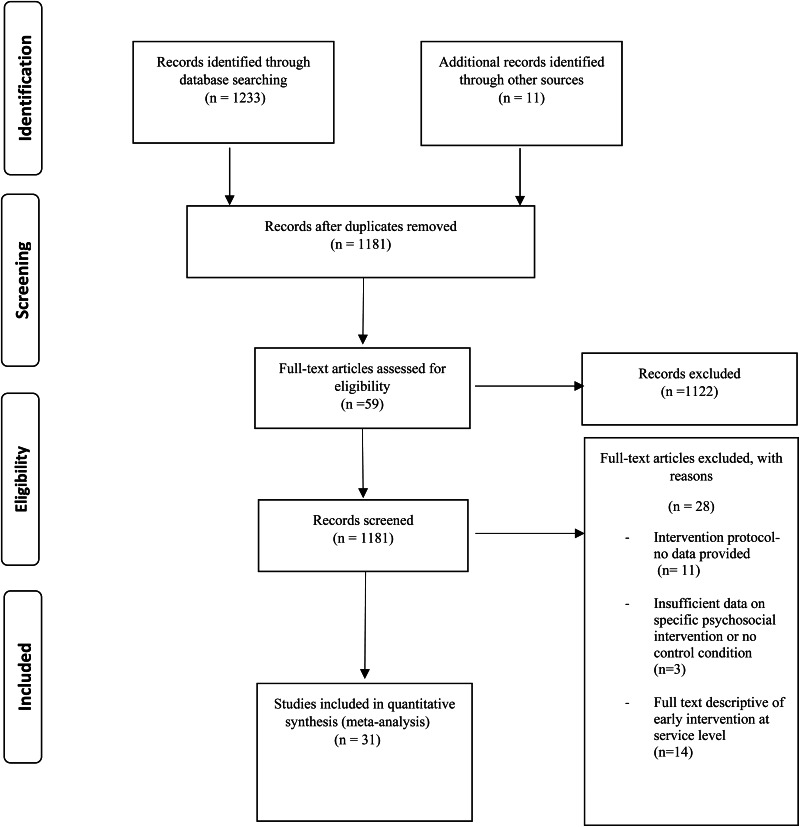
PRISMA flow diagram of studies selected for systematic review and meta-analysis.

The literature search identified 1233 relevant publications of which 20 were found to meet inclusion criteria. A further 11 studies were identified through additional sources. In total, 31 studies involving 2811 participants were included in our analysis (see Fig. 1 for PRISMA flow diagram and Table 1 for study characteristics). Studies are categorised by psychosocial intervention type as follows: cognitive behavioural therapy (CBT), family-based therapy (FBT), supported employment, cognitive remediation training (CRT) and multi-component psychosocial interventions.

**Table 1.** Characteristics of studies included in the review and meta-analysis

Study	Participants *N*		Mean age (s.d.)	Intervention	Outcome measures	Main findings	
	Intervention group	Control group					
*Cognitive behavioural therapy*							
Addington et al. (2011)	19	16	20.8 (4.5)	CBTp	GAF SAS SIAS SFS	No sig. impact in function	
Bechdolf et al. (2007)	29	38	25.2 (5.3)	CBTp	SAS	Sig. improvement in SAS however no sig. diff. between CBTp and control condition	
Fowler et al. (2009)	33	38	27.8 (6.1)	SRT	Time use	No sig. impact on function in affective psychosis group Sig. improvement in function in non-affective psychosis group 25% of individuals with non-affective psychosis engaged in paid work in the year following end of SRT	
Fowler et al. (2018)	72	71	24.8 (8.3)	SRT	Time use	SRCBT→increase of 8.1 h in structured activity	
Ising et al. (2016)	80 (UHR)	90	22.7 (5.6)	CBTp	SOFAS	No sig. impact in function	
Jackson et al. (2007)	31	31	22.5 (3.9)	CBTp	SOFAS	No sig. impact in function	
Morrison et al. (2004)	97 (UHR)	98	22.0 (4.5)	CBTp	GAF	No sig. impact in function	
Stain et al. (2016)	17 (UHR)	17	16.5 (3.2)	CBTp	GAF SOFAS	No sig. impact in function	
*Family-based intervention*							
Granö et al. (2016)	28 (UHR)	28	15.5 (1.6)	Family based intervention	GAF	Improvement in functioning	
Mcfarlane et al. (2015)	147 (CHR)	57	16.4 (3.3)	Family based intervention	GAF GFR GFS	Improvement on GAF, work, and school participation	
Miklowitz et al. (2014)	55 (CHR)	47	17.4 (4.1)	Family based intervention	GAF	>19 years: >improvement in GAF if received family intervention 16–19 years: >improvement in GAF if received control condition	
*Supported employment*							
Killackey et al. (2008)	20	21	21.3 (2.4)	IPS	Employment SOFAS	IPS>sig. higher employment and reduced welfare benefits	
Killackey et al. (2019)	66	60	20.4 (2.4)	IPS	Employment SOFAS	IPS>sig. higher employment (71%)	
Rosenheck et al. (2017)	144	83	23.2 (5.2)	SEE-supported employment and education	Participation in work or school	EI associated with > increase in participation in work or school and difference appeared to be mediated by SEE	
*Cognitive remediation*							
Choi et al. (2016)	30 (UHR)	32	18.3 (3.7)	CRT-process speed training	SAS	Improvement in function	
Eack et al. (2009/2010)	31	27	25.9 (6.3)	CRT-neurocognition + social skills group	SAS Major Role Inventory GAS	Improvement in social functioning/maintained at 1 year follow-up	
Lee et al. (2013)	18	18	22.8 (4.3)	CRT-neurocognition programme + psychoeducation	SFS	CR→improvement in function	
Loewy et al. (2016)	50	33	17.8 (3.1)	CRT-auditory processing programme	GAF GFS GFR	Improvement in function	
Østergaard Christensen et al. (2014)	51	47	25.0 (3.3)	CRT-neurocognition programme	UPSA-B	No. sig. impact on function	
Piskulic et al. (2015)	18 (CHR)	14	19.7 (5.7)	CRT – auditory training	GFS GFR	Sig. improvement in social function	
Ventura et al. (2017)	38	40	21.5 (3.0)	CRT-neurocognition programme + group	SAS	Sig. improvement in social functioning	
Vidarsdottir et al. (2019)	25	24	24.2 (3.2)	CRT-neurocognition programme + SCIT group	LSP-39 OSA Brief A	No sig. impact on function	
Wykes et al. (2007)	21	19	18.2 (2.5)	CRT – Neurocognition + TAU	SBS	Sig. impact on function	
Mendella et al. (2015)	16	11	25.0 (3.9)	CCT-Compensatory cognitive training	UPSA-B	No sig. impact on function	
*Multi-component psychosocial intervention*							
Albert et al. (2016)	30 (CHR)	29	26.6 (4.4)	Family treatment, social skills training, assertive treatment approach	SPS	No sig. impact on function	
Macneil et al. (2012)	20	20	21.8 (2.1)	CBTp, family therapy, psychoeducation	GAF SOFAS	Sig. improvement in functioning	
Palma et al. (2019)	35	27	25.5 (4.8)	CBTp, psychoeducation, cognitive-motivational therapy	GAF	Sig. improvement in functioning	
Penn et al. (2011)	22	22	23.5 (3.9)	CBTp, psychoeducation, motivational interviewing, social skills	RFS SSPA	No sig. impact on function	
Ruggeri et al. (2015)	239	153	29.3 (9.8)	CBTp, family, intervention, case management	GAF	Sig. improvement in functioning	
Schlosser et al. (2018)	38	21	24.3 (2.6)	Mobile application- community peer support, CBTp, goal setting	MAP-SR RFS	Trend towards sig. diff. on MAP-SR No sig. diff. on RFS	
Wessels et al. (2015)	31 (CHR)	43	25.2 (5.4)	CBTp, psychoeducation,	GAF	Sig. improvement in function.	

Brief-A, Behaviour Rating Inventory of Executive Function; GAF, Global Assessment of Functioning Scale; GAS, Global Assessment Scale; GFR, Global Functioning: Role Scale; GFS, Global Functioning: Social Scale; LSP-39, Life Skills Profile; OSA, Occupational Self-Assessment; MAP-SR, Motivation and Pleasure-Self Report scale; RFS, Role Functioning Scale; SAS, Social Adjustment Scale; SBS, Social Behaviour Schedule; SFS, Social Functioning Scale; SIAS, Social Interaction Anxiety Scale; SOFAS, Social & Occupational Functioning Scale; SSPA, Social Skills Performance Assessment; UPSA-B, UCSD Performance-Based Skills Assessment.

**Fig. 2.**
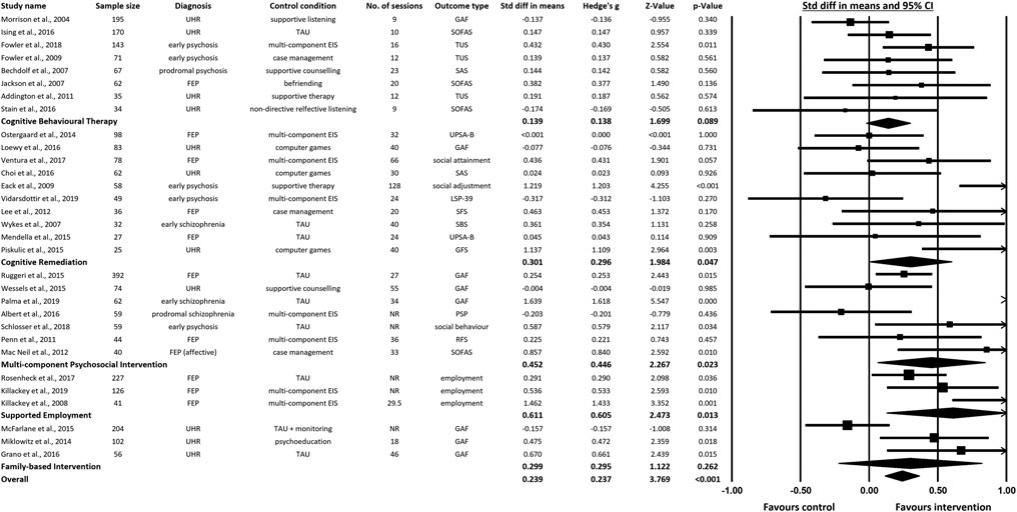
Forest plot of summary statistics (SMD – Cohen's *d*) for intervention groups and overall summary statistics for psychosocial interventions.

Meta-analysed results for all intervention categories for which relevant data could be ascertained are presented in Fig. 2 in terms of both the total effect and the effects of individual interventions where these could be estimated (*n* studies = 31). In summary these studies included 11 based on ultra-high-risk participants (*n* = 1040), two studies based on prodromal patients (*n* = 126), 11 first episode psychosis (FEP) studies (*n* = 1171), and a further seven studies of early psychosis (less than or equal to 5 years since diagnosis) (*n* = 474). Participants mean age ranged from 15.5 to 29.3 years (mean = 22.3, s.d. = 3.6). Mean percentage of male participants across studies was 63.3%. Across these, 22 studies included measures of global function (GAF, SOFAS, TUS, SAS, SFS, RFS, PSP and LSP-39) four studies included measures of social functioning (GFS, SBS, social behaviour and social attainment), three studies included measures of employment, and two studies included a measure of functional capacity (UPSA-B). For a description of validated functioning measures see online Supplementary Table S1. Intervention length ranged from 2 months to 3 years (mean = 8.7, s.d. = 7.7). Number of sessions ranged from 9 to 128 (mean = 32.1, s.d. = 24.2).

Across the total number of studies included, an effects size SMD = 0.239 [95% CI (0.115–0.364), *p* < 0.001] was observed, suggesting a benefit of psychosocial interventions generally in terms of social and occupational outcomes (see Fig. 2). When non-ramdomised controlled trial (RCT) studies (Granö et al., 2016; Macneil et al., 2012; Mcfarlane et al., 2015) were excluded from the analysis the effect size SMD changed to 0.251 (Granö et al., 2016; Macneil et al., 2012; Mcfarlane et al., 2015) (see online Supplementary Fig. S11).

Significant heterogeneity was noted for all intervention modalities, except for CBT (see online Supplementary Table S2). This is likely reflecting variability across studies in sample size, intervention length, number of sessions, participant diagnosis and outcome measures. For CBT, CRT and multi-component psychosocial interventions, no evidence of significant publication bias was found. Similarly, when all studies are considered together, no evidence of significant publication bias was observed. The limited number of studies in the supported employment and family-based intervention groups prevented publication bias from being thoroughly tested (see online Supplementary Figs. S1–S4).

### CBT in at risk and early psychosis

CBT for psychosis (CBTp) was developed with the primary aim of reducing clinical symptom severity and relapse rates, rather than to improve social and occupational function. Where social and occupational outcomes are reported, this is often as a secondary aim, if at all. CBTp is recommended by the National Institute for Health and Care Excellence for people living with a diagnosis of schizophrenia [National Institute for Health and Care Excellence (NICE), 2014]. A recent Cochrane review of CBTp concluded, however, that there remains a lack of robust evidence to support its clinical use in addition to standard care, on account of low-quality data available (Jones et al., 2018). Similarly, Bighelli et al. (2018) reported that while CBTp was associated with decreased positive symptoms, confidence in the estimates ranged from moderate to very low (Bighelli et al., 2018). Equally Laws, Darlington, Kondel, Mckenna, and Jauhar (2018) in their meta-analysis reported that CBTp has a small therapeutic effect on functioning at end-of-trial, but that this benefit did not persist at follow-up (Laws et al., 2018).

Based on our review of studies carried out in early psychosis, only six studies were identified that investigated the effects of CBTp – as a single-component intervention – on social and occupational functioning. Of these, five studies focused on clinical high-risk groups, none of which found evidence that CBTp was associated with improvements on the measures of social and occupational function, which was variously measured using the GAF, SAS, SFS, Time Use and the SOFAS (Addington et al., 2011; Bechdolf et al., 2007; Ising et al., 2016; Morrison et al., 2004; Stain et al., 2016).

One study of CBTp targeted psychosocial function in first episode psychosis (Jackson et al., 2007). It compared CBTp to a befriending intervention and demonstrated no significant difference between intervention groups post treatment (Jackson et al., 2007). Several additional studies included CBTp as one component of a multicomponent intervention; these are reviewed below in the section on multicomponent psychosocial interventions.

One question raised by these findings is whether a failure to see improvements in social and occupational function derives from a failure to ameliorate clinical symptoms, or whether successful improvement of clinical symptoms simply was not associated with any effects on functional outcomes. This question reflects a broader critique of CBTp in which the ability of CBT to lead to improvements in clinical state has been questioned (Fusar-Poli et al., 2015; Jones et al., 2018; Laws et al., 2018; Velthorst et al., 2015). Of the four studies above, however, each reported evidence that CBTp led to lowered symptoms, particularly positive symptoms, in the absence of a knock-on benefit to social and occupational function (Addington et al., 2011; Ising et al., 2016; Morrison et al., 2004). This was not always superior to the control condition (Stain et al., 2016). Ising et al. (2016) further stating social functioning remained impaired even in those remitted from ultra-high-risk status (Ising et al., 2016).

Other approaches to CBT for psychosis have emerged over time, shifting focus from symptoms to specifically targeting social recovery. In such approaches, the emphasis is on addressing barriers to social engagement (e.g. avoidance), and participation in normative life roles. Although there is much overlap with traditional CBTp in terms of collaborative formulation and goal setting, a stronger emphasis is placed on behavioural experimentation outside the clinic and in the person's own social environment to overcome identified barriers. Described as social recovery therapy (SRT), this approach has been demonstrated to lead to significant improvement in function as measured by time spent in structured activity. Importantly, this approach also showed evidence of improvements being maintained over time when compared to a control group receiving treatment a usual (Fowler et al., 2009, 2018; Fowler, Hodgekins, & French, 2019). The degree to which these changes were independent of changes in symptom severity is unclear; missing data on symptoms severity at follow-up assessment time points has meant that this question remains to be answered.

A meta-analysis of the effects of CBT interventions on validated measures of function was non-significant based on a total of eight available studies [SMD = 0.139, 95% CI (−0.021 to 0.299), *p* = 0.089] (see Fig. 2). Five of these eight studies were based on ultra-high risk (UHR) samples. Of note, when the analysis was conducted excluding those UHR studies a difference in effect size and significance was observed (SMD = 0.345, *p* < 0.005) (see online Supplementary Fig. S12). Although only three studies were included in this additional analysis, it is an important exploratory consideration.

Also noteworthy in the CBT intervention group was that the largest of these studies – based on SRT rather than a symptom orientated CBT, was the sole individual study associated with significant gains in psychosocial function (Fowler et al., 2018).

### Family-based interventions

Family interventions are recommended by the National Institute for Health and Care Excellence (NICE) clinical guidelines in the treatment of early psychosis [National Institute for Health and Care Excellence (NICE), 2015]. Those at-risk for or in the early stages of psychosis often continue to live with and be supported by family members in the community. It is widely acknowledged that this experience impacts not only on the individual, but also on family members in terms of their daily functioning, relationships, mental health and community interaction.

Family intervention has typically focused on relapse prevention, often by seeking to enhance communication and problem solving within the family to reduce expressed emotion, stress and the consequent risk of relapse. Only three studies were identified that reported the effects of family therapy on social and occupational function when delivered as a sole intervention (by comparison with multi-component studies reviewed below). Each of these studies focused on clinical high-risk groups. Although the content of family intervention delivered in each study varied, key common elements of each included psychoeducation, communication skills and problem solving for everyday living. Similar to SRT, delivering family therapy as part of, or embedded in, community activities (directly in natural setting of the participant e.g. meeting in a café) featured in two of the studies and described as ‘assertive community treatment’, or ‘community-orientated integrative treatment’ (Granö et al., 2016; Mcfarlane et al., 2015). All three studies report a significant improvement in function based on based on both measures of social function and levels of participation in normative life activities such as school or work (Granö et al., 2016; Mcfarlane et al., 2015; Miklowitz et al., 2014). One study further compared the impact of family therapy on psychosocial functioning between those over and under the age of 19, with a stronger treatment effect reported in those over the age of 19 (Miklowitz et al., 2014).

In terms of whether and how these effects related to changes in clinical presentation, two of the three studies reviewed report a significant reduction in symptoms, particularly positive symptoms concurrent to psychosocial improvements (Mcfarlane et al., 2015; Miklowitz et al., 2014). The third study reviewed reported improvement in psychosocial functioning, self-reported depression symptoms and hopelessness in the absence of changes in either self-reported anxiety, or psychosis risk symptoms as measured by the Structured Interview for Psychosis-Risk Syndromes (SIPS) (Granö et al., 2016).

An insufficient number of family therapy studies (*n* = 3) were available to calculate an effects size specifically for family interventions. When reviewed in the overall list of psychosocial studies (online Supplementary Fig. S3), effect sizes differed between studies, with Granö et al. (2016) and Miklowitz et al. (2014) showing significant psychosocial benefits, while the study by Mcfarlane et al. (2015) reported non-significant benefits (Granö et al., 2016; Mcfarlane et al., 2015; Miklowitz et al., 2014). Of note also in the FBT group is that although all three studies included a control condition, both Granö et al. (2016) and Mcfarlane et al. (2015) are not randomised control trials and this also needs to be considered in the interpretation of the exploratory results.

### Supported employment

Individuals with lived experience of psychosis often report they place goals of completing their education and gaining employment above addressing their mental health symptoms (Ramsay et al., 2011). Despite these stated goals, the trajectory of young people living with psychosis to complete their education and transition into employment remains low (Rinaldi et al., 2010; Waghorn et al., 2012). Under the umbrella of the supported employment model, the individual placement and support (IPS) model has been integrated into clinical guidelines and several early intervention services and represents a research focus of studies of psychosocial function in early psychosis [National Institute for Health and Care Excellence (NICE), 2015]. IPS is designed to assist people with severe mental illness to return to mainstream employment, the overarching philosophy being that anyone is capable of partaking in paid, competitive employment with careful consideration of job type, job environment and with an effective support system in place.

IPS is based on eight key principles; zero exclusion, individual job preferences, a goal of competitive employment, employers are approached with the needs of the individual in mind, provision of ongoing time-unlimited support, integration within the mental health treatment team, job search begins directly on entry into the IPS programme, and personalised benefits counselling. IPS is typically provided as part of a wider early intervention service, making the disentanglement of the effect on function difficult. Moreover, intervention components of IPS overlap to an extent with SRT and FBT in terms of psychoeducation, problem solving skills, goal formulation and notably a community-based, practical approach to recovery.

We identified three studies reporting on supported employment in early psychosis with no studies identified in relation to the clinical high-risk group. Two IPS studies in first-episode psychosis reported a significant impact on function as measured by participation in employment and reduced utilisation of welfare benefits (Killackey et al., 2019; Killackey, Jackson, & McGorry, 2008). Unlike CBTp and FBT studies discussed in this review, clinical presentation, and the impact of IPS on symptom severity were not reported in these studies. Instead, the studies focused on whether recovery of social and occupational functioning was maintained over time. In particular, these studies focused on whether return to work and gains in educational attainments and were sustained over time when compared to early intervention services where staff are upskilled in vocational recovery. Similarly, a third early psychosis study took education into account, reporting on a supported employment and education intervention, informed by the broader supported employment model and IPS, combined with supported education services (Rosenheck et al., 2017). This intervention was provided in the context of an early intervention service. They report increased participation in work or school, which appears to be mediated, in part, by the supported education service.

Similar to FBT, there was an insufficient number of IPS-based studies from which to generate an intervention-specific effect size. However, as online Supplementary Fig. S3 illustrates, the three studies included in our overall meta-analysis showed significant effects favouring the intervention groups.

### Cognitive remediation training (CRT)

CRT is a ‘behavioural training-based intervention which aims to improve cognitive processes (attention, memory, executive function, social cognition or metacognition) with the goal of durability and generalisation’ [‘Cognitive Remediation Experts Workshop (CREW)’, Florence, April 2010]. For schizophrenia generally, a meta-analysis of CRT reported an effect size of Cohen's *d* = 0.45 for cognitive performance, *d* = 0.42 for psycho-social functioning and *d* = 0.18 for symptom severity. Wykes, Huddy, Cellard, Mcgurk, and Czobor (2011) further concluded that CRT is more effective when provided in the context of a rehabilitation setting, allowing individuals to put their training into practice (Wykes et al., 2011). Different CRT interventions targeted a variety of perceptual and cognitive skills, including social cognition (e.g. emotion processing or facial affect recognition) with the goal of translating training into improved social and occupational functioning. A meta-analytic investigation of social cognitive training for schizophrenia in 2012 demonstrated moderate to large effects on observer-rated community and institutional function (Cohen's *d* = 0.78) (Kurtz & Richardson, 2012). One criticism of CRT has been the high level of the 1:1 therapy time involved. However, we have reported evidence that significant improvements in both neuropsychological function and social/occupational functioning following a computer-based working memory intervention that required only weekly 1 h 1:1 support (Donohoe et al., 2018).

What is the evidence for impact of CRT on social and occupational functioning in the clinical high-risk and early psychosis groups? Our review identified 10 studies providing a CRT intervention reporting on validated measures of function in these groups. Two studies reported on CRT in the UHR group. Piskulic, Barbato, Liu, and Addington (2015) report significant improvement in function in the intervention group while Choi et al. (2016) report a non-significant impact. Interestingly, both studies were computer-game based with a primary cognitive outcome however varied in terms of the intervention setting and type of functional outcome used. Piskulic et al. (2015) were delivered online and utilised a social functioning measure while Choi et al. (2016) were delivered in a traditional clinic setting and used a global measure of function. This will be considered further in the discussion below.

Five of eight studies in the early psychosis group reported evidence of a significant impact on social and occupational functioning outcomes. Of note, each of these interventions included components such as psychoeducation or a social skills group that scaffolded training e.g. by specifically relating it to greater social involvement (Eack et al., 2009; Eack, Greenwald, Hogarty, & Keshavan, 2010; Lee et al., 2013; Loewy et al., 2016; Ventura et al., 2017).

The remaining three studies in the early psychosis group reported no significant effect of the CRT intervention on psychosocial functioning (Mendella et al., 2015; Østergaard Christensen et al., 2014; Vidarsdottir et al., 2019). In the Østergaard Christensen et al. (2014) study a failure to observe benefits to psychosocial function was despite improvements in symptom severity, cognitive function and self-esteem (Østergaard Christensen et al., 2014). Vidarsdottir and colleagues report no improvement in either symptoms or social functioning (Vidarsdottir et al., 2019). Similarly, Mendella et al. (2015) report improvements in cognitive domains but no impact on psychosocial functioning or symptoms (Mendella et al., 2015). An interpretation of these findings is that although all the above studies found evidence of improved cognitive function following CRT, these benefits were more likely to translate to benefits in social and occupational function when delivered alongside additional components that promoted broader recovery and greater psychosocial engagement. In short, as with CRT interventions delivered in chronic schizophrenia (SZ), CRT in early psychosis is more likely to be beneficial when provided in the context of broader rehabilitation (e.g. early intervention services).

The data from the 10 CRT studies were available for meta-analysis, allowing us to test the significance of this intervention separately. As illustrated in Fig. 2, CRT was associated with modest but significant improvements in social and occupational function when compared to control conditions [SMD = 0.301, 95% CI (0.004–0.599), *p* = 0.047]. As illustrated by Fig. 2, difference in effect sizes reported could not be easily understood in terms of differences in sample type (first-episode/early psychosis groups *v.* UHR groups).

### Multi-component psychosocial intervention

The concept, purpose and effectiveness of multi-component early intervention for psychosis services (EIS) has recently been described in a meta-analysis (Correll et al., 2018). As described by Correll et al. (2018) these interventions included the ‘core’ components of psychopharmacological treatment (with regular medication review) and family psychoeducation/counselling, alongside ‘optional’ components of CBT, family therapy, vocational and education counselling, social skills training, crisis management and a crisis response team. The range of intervention components was 4–6 with a mean of 4.8 (0.9) components. Important clinical outcomes in this study were considered as all-cause treatment discontinuation, hospitalisation, total and specific (positive, negative, general and depressive) symptom severity, global functioning and involvement in school or work and quality of life (Correll et al., 2018). The authors report superior outcomes for all 13 meta-analysable outcomes over treatment as usual (TAU) at several time points of treatment with small to moderate effect sizes evident. In terms of social and occupational functioning, seven studies (*n* = 1005) reported global functioning improving significantly more in EIS than TAU with six studies (*n* = 1743) also reporting significantly higher participation in school or work in EIS than TAU.

In our review of psychosocial interventions, we reviewed those studies that estimated the effects on psychosocial function of multi-component psychosocial intervention. Specifically, here, multi-component psychosocial intervention refers to studies which incorporate more than one psychosocial treatment approach from among CBTp, social skills training, family training and psychoeducation, but without the explicit inclusion of a pharmacological intervention, medication review or stipulation of core or fundamental components. In short, although it is acknowledged pharmacotherapy is frequently offered, these multi-component psychosocial interventions, rather than providing a single therapeutic approach, apply several approaches and underlying therapeutic principles with the aim of improving social and occupational functioning. Seven studies were identified under this category (see online Supplementary Table S2), two based on in high-risk samples and five based on individuals with early psychosis. Of the high-risk studies, Albert et al. (2016) found no evidence of improvement, despite observing that low levels of functioning were a consistent predictor of transition to psychosis (Albert et al., 2016). By comparison, Wessels et al. (2015) reported evidence of significant increase in function (as measured by the GAF scale) following a multi-components intervention (Wessels et al., 2015).

In the early psychosis group, four of the five studies report improvement in functioning in early psychosis (Macneil et al., 2012; Palma et al., 2019; Ruggeri et al., 2015; Schlosser et al., 2018). Intervention approaches in this category had the consistent features of adopting a manualised approach to the components provided and selecting individual intervention components based on the specific patients. The flexibility of intervention component selection in particular appears beneficial to individual and group outcomes in terms of psychosocial functioning; heterogeneity between these manualised approaches may present challenges in terms of replication of results and direct comparison between studies.

The data from the seven multi-component psychosocial studies were also available for meta-analysis. Figure 2 illustrates this group was also associated with modest but significant improvements in social and occupational functioning when compared to a control condition [SMD = 0.452, 95% CI (0.061–0.843), *p* = 0.023]. When the non-RCT study (Macneil et al., 2012) in this intervention category was excluded from the analysis the effect size SMD changed to SMD = 0.395) (see online Supplementary Fig. S11).

### Meta-analysis by illness stage, length and duration of intervention, and outcome measurement type

Subgroup analyses were performed to compare effect sizes based on diagnosis, length of intervention, number of sessions, control condition, mode of delivery and type of outcome measure (see online Supplementary Figs. S5–S10). When compared for diagnosis (UHR *v.* FEP *v.* early psychosis), the SMD was largest for the early psychosis group [SMD = 0.572, 95% CI (0.129–1.014), *p* = 0.011], followed by the FEP group [SMD = 0.360, 95% CI (0.198–0.521), *p* < 0.001], and the smallest effect size was found for UHR group [SMD = 0.107,95% CI (−0.066 to 0.280), *p* < 0.001]. For length of intervention, studies with duration of 6 months or less were compared to those with duration of greater than 6 months. Effect sizes were larger for studies with a longer duration [SMD = 0.397, 95% CI (0.149–0.645), *p* = 0.002] compared to studies of 6 months or less [SMD = 0.251, 95% CI (0.088–0.415), *p* = 0.003]. Similarly, when compared based on number of sessions, studies with >30 sessions showed a larger effect [SMD = 0.487, 95% CI (0.158–0.816), *p* = 0.004] than those with 30 sessions or less [SMD = 0.225, 95% CI (0.077–0.372), *p* = 0.003]. For control condition, studies that used an active control showed a smaller effect [SMD = 0.258, 95% CI (0.091–0.424), *p* = 0.002] than those that compared the intervention to TAU [SMD = 0.464, 95% CI (0.194–0.733), *p* < 0.001]. For mode of delivery of the intervention, community-based interventions [SMD = 0.376, 95% CI (0.129–0.623), *p* = 0.003] showed a larger effect than clinic-based interventions [SMD = 0.264, 95% CI (0.081–0.447), *p* = 0.005]. Interventions delivered online showed the largest effect size [SMD = 0.497, 95% CI (−0.179 to 1.174), *p* = 0.150], however this effect was not significant and was based on only three studies. Finally, studies were grouped based on type of outcome measure used – we compared measures of general function to more specific measures of function (global function *v.* social function *v.* employment). There was a notable difference in effect size between these groups. Results of this subgroup analysis showed much larger effect sizes for studies that used more specific measures of employment [SMD = 0.611, 95% CI (0.127–1.095), *p* = 0.013] or social functioning [SMD = 0.716, 95% CI (0.372–1.060), *p* < 0.001] compared to global functioning measures [SMD = 0.197, 95% CI (0.049–0.346), *p* = 0.009].

## Discussion

This review and meta-analysis focused on psychosocial interventions that sought to improve social and occupational function in the early stages of psychosis, a relatively recent and emerging focus of psychosis research. Previously, psychosocial interventions had focused either solely, or principally on reducing clinical symptoms severity as their endpoint, on the basis that this would be associated with improved functional outcomes. The absence of empirical support for this expectation has in large part informed this wider focus on and targeting of social and occupational function. As reviewed here, studies that have taken up this challenge have been varied in terms of intervention, outcomes measured used, and participants. Notwithstanding this heterogeneity, broad evidence was observed to support the efficacy of psychosocial interventions for improving social and occupational function in the early stages of psychosis.

In addition to this general conclusion, several specific conclusions can also be made. Firstly, our narrative review of the available evidence suggests that delivering psychosocial intervention in community-based (rather than clinic-based settings) settings is a key consideration. Community-based, assertive outreach approaches – irrespective of treatment type – appear to have a greater impact on function in the early psychosis population. Moving from clinic-based interventions towards providing treatment in the person's usual environment with involvement of key community stakeholders appears a key ingredient for effectiveness and collaborative, patient-centred working. For example, when compared to CBTp studies, where clinical improvement was not necessarily associated with improved social functioning, family-based intervention studies reporting evidence of improvement in social and occupational functioning in the clinical high-risk group tended to also report evidence of improvement in clinical presentation. One possible factor in this difference in social and occupational outcomes was the setting, with family-based interventions more likely to be delivered in the community, outside a traditional clinic setting. As noted above, social recovery orientated CBT, which is employs an assertive outreach approach and is delivered in a community setting was also found to be effective in improving social and occupational functioning (see online Supplementary Fig. S9).

Secondly, a personalised approach to treatment that matches the psychosocial interventions provided to the needs of the individual appears critical to meeting the complex needs of individuals in the early stages of psychosis. Multi-component interventions (both at an early intervention service level and psychosocial intervention level), tailored to the needs of the individual, appear to have greater potential to impact a range of psychosocial treatment targets. Critical to the success of these multi-component interventions would appear to be the capacity to provide these components flexibly in a manner adapted to the changing needs and circumstances of individual.

In estimating the contribution of individual psychosocial intervention types, both treatment intensity and duration were observed to moderate efficacy. As noted in the findings of our meta-analysis, interventions of a 6-month duration or longer or >30 sessions were found to have a greater impact on social and occupational functioning when compared to those 6 months or less or <30 sessions (see online Supplementary table and Figs. S6 and S7).

Similarly, the type of measurement used when considering social and occupational function was observed to significantly influence the size of effect observed, with measures that specifically targeted social functioning and engagement, and employment activity yielding a more sensitive estimate of change following intervention that more global indicators (see online Supplementary Fig. S9).

Furthermore, stage of illness – whether pre or post first diagnosis of psychosis was also observed to impact on the efficacy of treatments. In particular, improvement in psychosocial function following the interventions reviewed was greater for individuals following a diagnosis of psychotic illness (FEP or early psychosis compared to UHR). This evidence may reflect the fact that a further decline in psychosocial function following diagnosis creates a wider target for the interventions considered here to have an effect. If true, we speculate that this may not mean that interventions targeting psychosocial function are less effective in the UHR or FEP group, but simply that level of social and occupational function continues to decrease during this time, thus creating a larger window of deficits in which to demonstrate recovery. This finding is considered in the context of the review limitation of the variability in defining stage of illness across studies, the impact on recruitment and inclusion criteria of individual studies, and the clinical heterogeneity of the UHR group.

As noted, a review and meta-analysis on the impact of psychosocial intervention on validated measures of function is an emerging area of research in the area of early psychosis. This study, although providing preliminary evidence of effectiveness of psychosocial intervention in this area, is not without its limitations. Firstly, we note the heterogeneity in study design and methodologies in this area of research. The quality evaluation scale (Rokita et al., 2018) employed for this review while meeting quality assurance standards did not account for variation in randomisation and blinding and this should be considered in future reviews and meta-analyses.

A second consideration is the heterogeneity of validated measures of function ranging from global assessments to individual measure of function. The authors conclude how they measure function and considering social and occupational functioning as a primary outcome in the early psychosis group is a priority consideration in future study design. This will have potential impact on study replicability, and comparison of high-quality psychosocial intervention studies at a meta-analytical level.

Thirdly, the authors also acknowledge the lack of available data in the included studies in terms of the acceptability of the intervention to the participants and also the adherence to therapy during individual studies. Monitoring adherence to TAU, including pharmacotherapy, is also vital in future study design. These are priority considerations for future research and are likely not only to contribute to the quality of future studies but also the translation to clinical practice.

In conclusion, the increased emphasis on the value of targeting and treating social and occupational function in the early treatment of psychosis appears to be well founded. As reviewed here, there is evidence that many, but not all, psychosocial interventions are associated with improvements in these areas. We emphasise that the findings from two of the included intervention groups (FBT and IPS) are exploratory in nature due to the small number of studies included. However, we highlight that CRT, multi-component psychosocial intervention and CBT (with an emphasis on assertive outreach) emerge as providing robust evidence for clinical implementation in the early psychosis group. Providing these as part of multi-component interventions in community-based settings remains an important need for this cohort. Supporting the recent progress in increasing the availability of these interventions remains a key priority.

## Supplementary material

For supplementary material accompanying this paper visit https://doi.org/10.1017/S003329172100341X. click here to view supplementary material
