# Supplementary material for: Social and occupational recovery in early psychosis: a systematic review and meta-analysis of psychosocial interventions
Source: Psychol Med. 2021 Sep 3;53(5):1787–98. doi: 10.1017/S003329172100341X (PMC10106304; doi:10.1017/S003329172100341X)
Supplement: Supplementary file 1 [file S003329172100341Xsup001.docx]

**Table 1. Description of psychosocial functioning measures used across intervention studies.**

| Measure | Scoring | Description |
| --- | --- | --- |
| Global (social and occupational) functioning measures | | |
| Global Assessment of Functioning (GAF) | Scale: 0-100, higher scores indicate better function | Measures social, occupational, and psychological functioning |
| Social and Occupational Functioning Assessment Scale (SOFAS) | Scale: 0-100, higher scores indicate better function | Measures social, occupational, or school functioning |
| Social Functioning Scale (SFS) | Scale: 55-135, 79 item questionnaire, 7 subscales summed for total score, higher scores indicate better function | Measures social engagement/withdrawal, interpersonal behaviour, pro-social activities recreation, independence-competence, independence-performance, employment/occupation. |
| Social Adjustment Scale (SAS) | Scale: 0-5, 52 item questionnaire, lower scores indicate better function | Measures work, household, social activities/leisure, physical well-being, general adjustment summary score |
| Role Functioning Scale (RFS) | Scale: 4-28, 4 subscales rated 1-7, subscales summed for total score, higher scores indicate better function | Measures work productivity, independent living, immediate social network, and extended social network |
| Personal and Social Performance Scale (PSP) | Scale: 0-100, higher scores indicate better function | Measures socially useful activities including work and study, personal and social relationships, elf-care, and disturbing and aggressive behaviour |
| Life Skills Profile (LSP-39) | Scale: 38-156, 39 item questionnaire, lower scores indicate better function | Measures communication, social contact, non-turbulence, self-care, and responsibility |
| Time Use Survey (TUS) | Hours per week of structured activity | Structured activity includes paid and voluntary employment, education and training, childcare, housework and chores, leisure and sports, socialising. |
| Functional capacity measures |  |  |
| UCSD Performance-based Skills Assessment - Brief (UPSA-B) | Scale: 0-100, higher scores indicate better function | Measures ability to perform various tasks related to everyday functioning (e.g., communicating by telephone, counting money, paying bills) |
| Social functioning measures |  |  |
| Social Behaviour Schedule (SBS) | Scale: 0-84, 21 item questionnaire, lower scores indicate better function | Measures antisocial behaviour, depressed behaviour, social withdrawal and thought disturbance. |
| Global Functioning: Social Scale (GFS) | Scale: 1-10, higher scores indicate better function | Measures quantity and quality of peer relationships, level of peer conflict, age-appropriate intimate relationships, and involvement with family members |
| UCLA Social Attainment Survey | Scale: 0-5, higher scores indicate better function | Measures peer relationships, leadership in peer relationships, dating history, sexual experience, outside activities, and participation in organizations |

**Table 2. Heterogeneity analysis.**

| Fixed effect analysis | | | Heterogeneity | | | |  | Tau | | | |
| --- | --- | --- | --- | --- | --- | --- | --- | --- | --- | --- | --- |
| Group |  | **No. of studies** | **Q-value** | **df (Q)** | ***p*-value** | **I-squared** |  | **Tau-Squared** | **Standard Error** | **Variance** | **Tau** |
| CBT |  | 8 | 8∙448 | 7∙000 | 0∙295 | 17∙137 |  | 0∙009 | 0∙028 | 0∙001 | 0∙095 |
| CRT |  | 10 | 26∙580 | 9∙000 | 0∙002 | 66∙139 |  | 0∙148 | 0∙109 | 0∙012 | 0∙384 |
| MCI |  | 7 | 29∙859 | 6∙000 | 0∙000 | 79∙906 |  | 0∙211 | 0∙177 | 0∙031 | 0∙459 |
| SE |  | 3 | 6∙869 | 2∙000 | 0∙032 | 70∙882 |  | 0∙122 | 0∙188 | 0∙035 | 0∙349 |
| FBI |  | 3 | 9∙994 | 2∙000 | 0∙007 | 79∙988 |  | 0∙168 | 0∙216 | 0∙047 | 0∙410 |
| Overall |  | 31 | 88∙918 | 30∙000 | 0∙000 | 66∙261 |  | 0∙095 | 0∙042 | 0∙002 | 0308 |


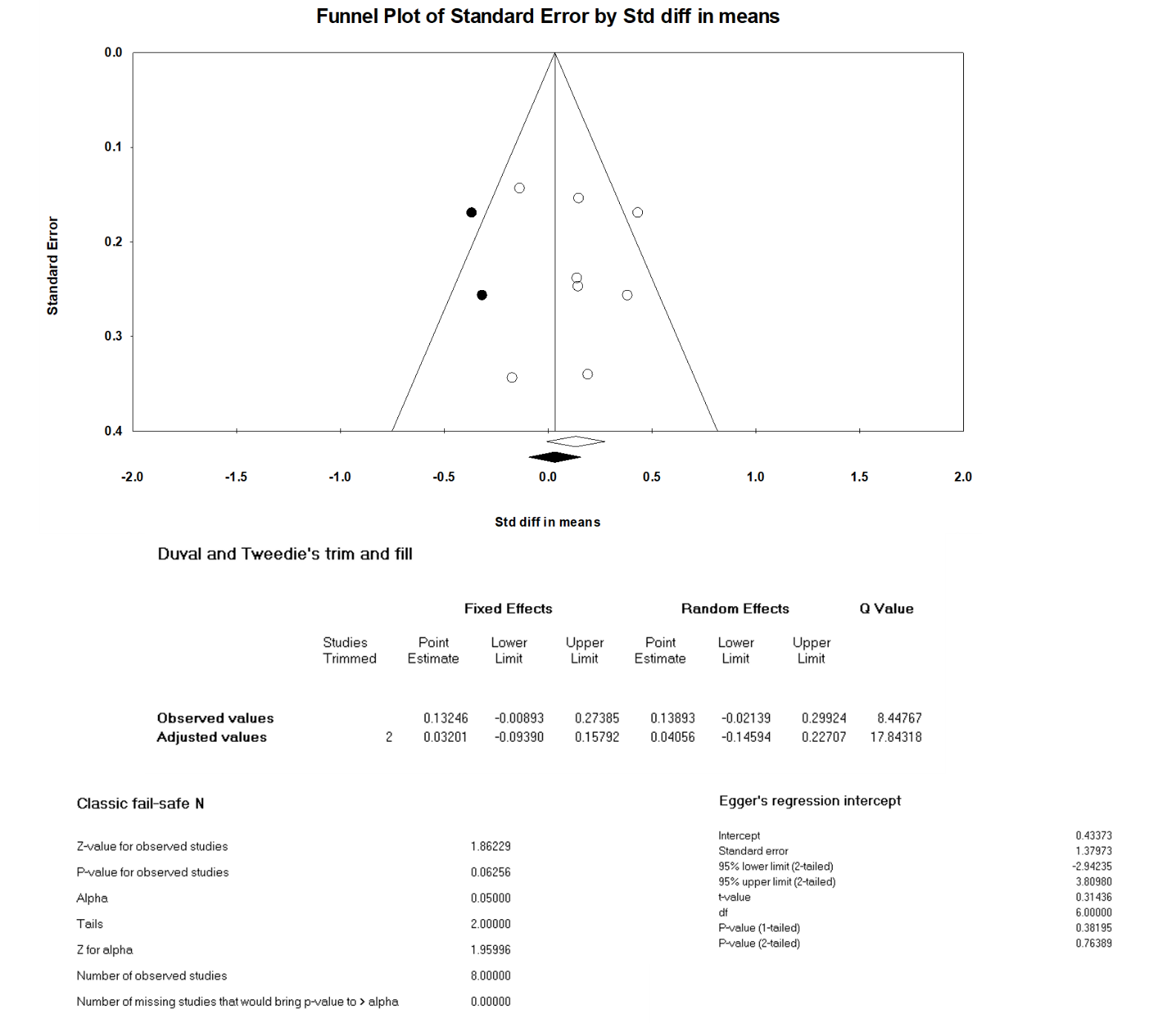


**Figure 1. Publication bias: CBT**


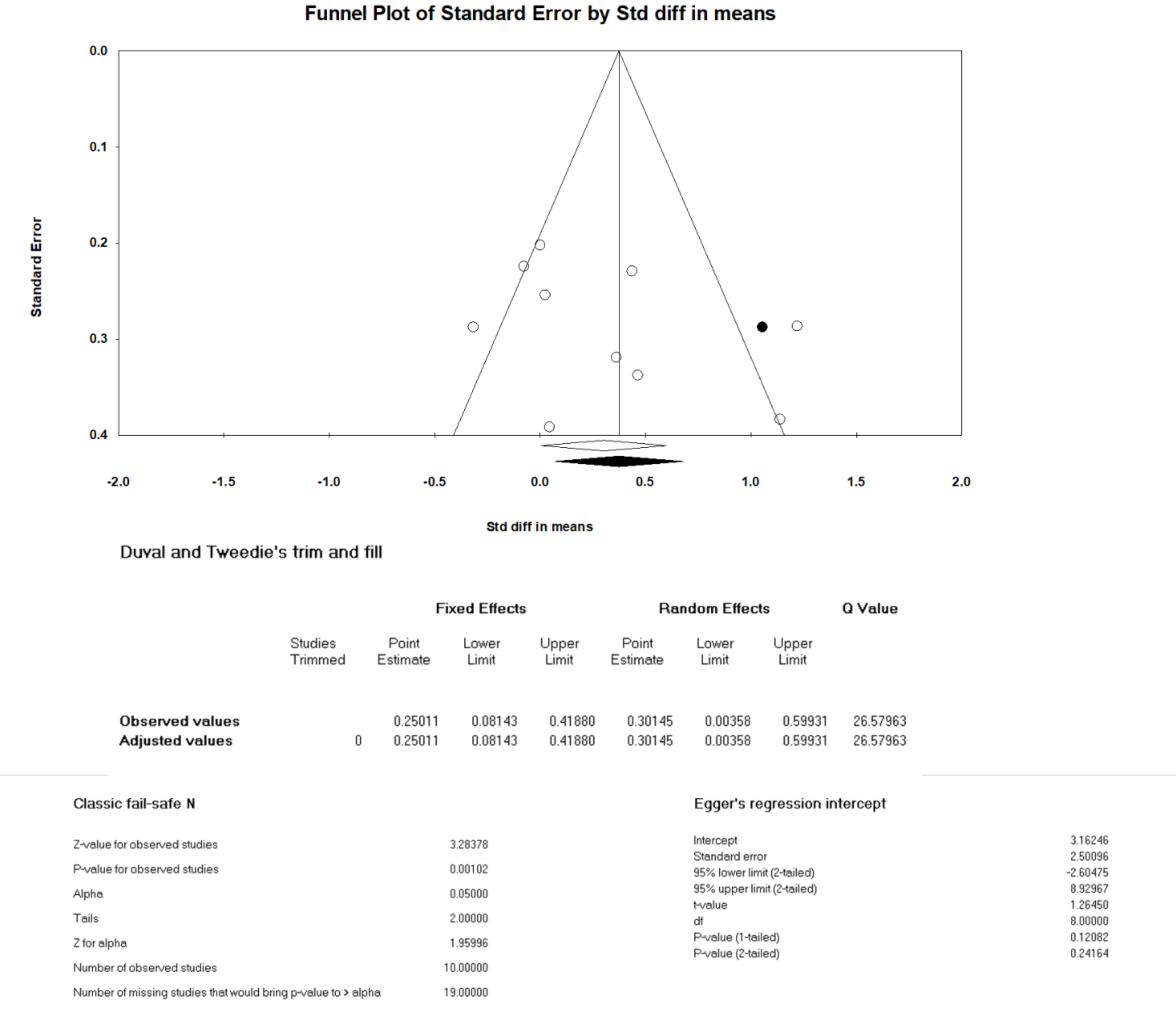


**Figure 2. Publication bias: CRT**


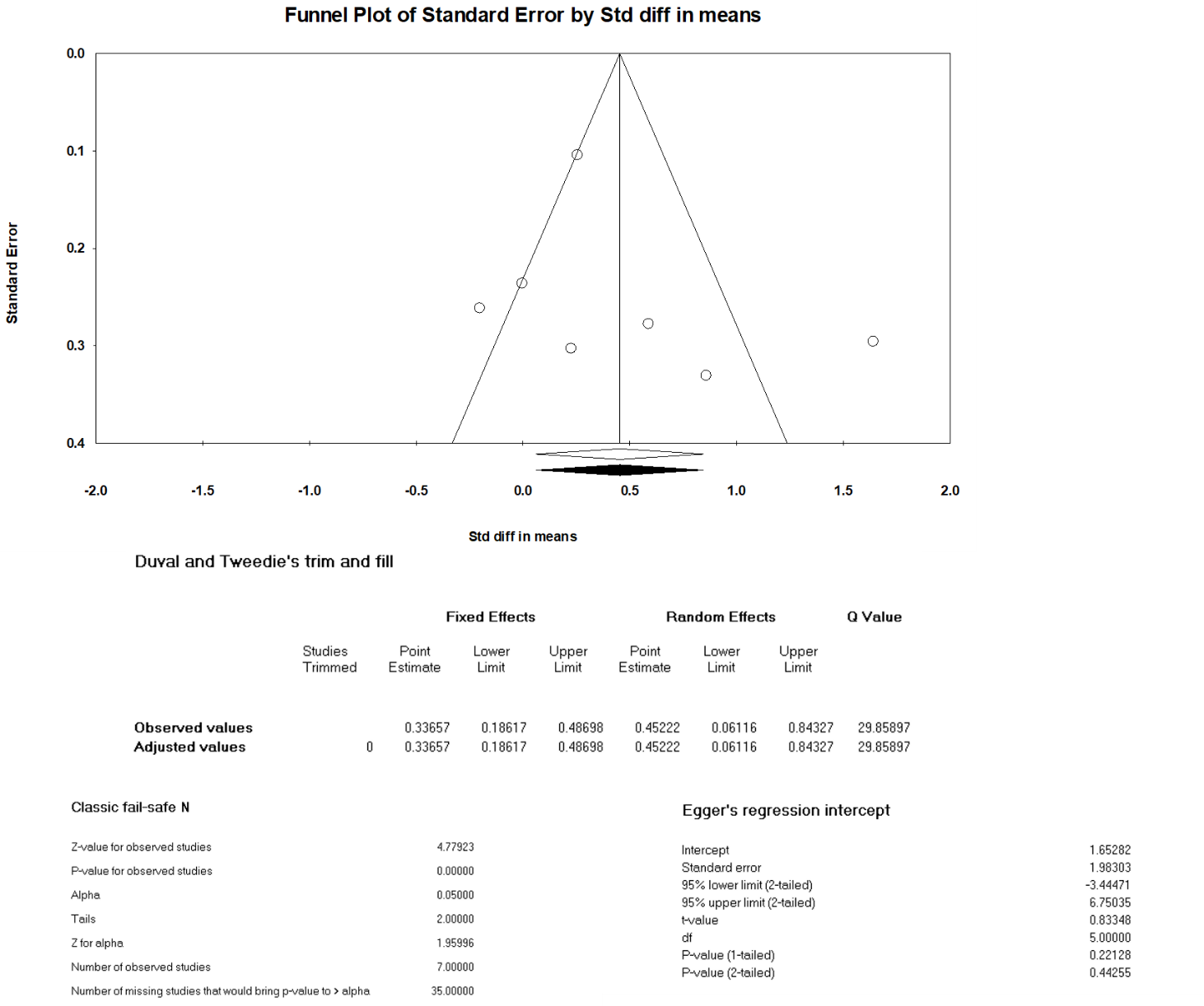


**Figure 3. Publication bias: MCI**


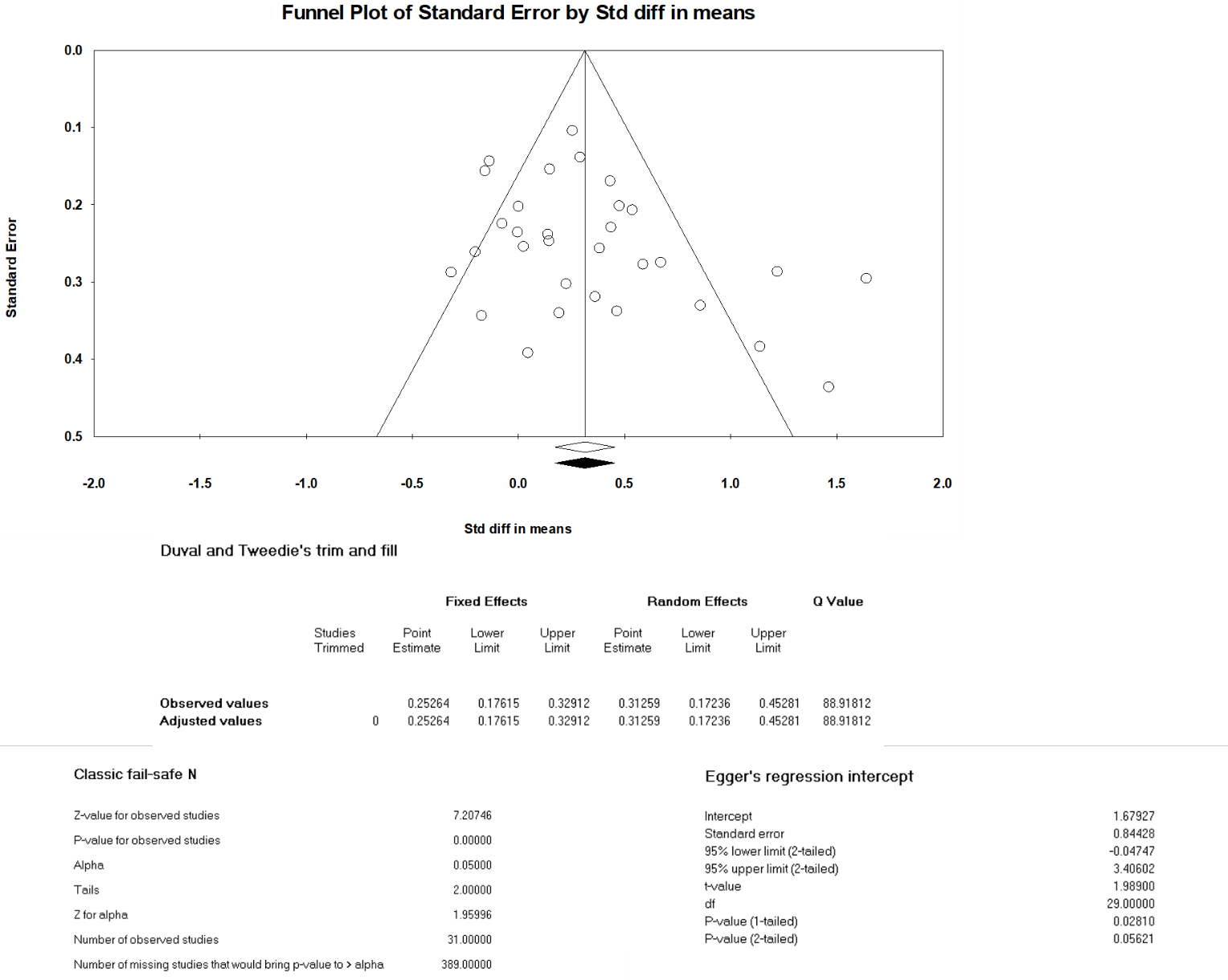


**Figure 4. Publication bias: overall psychosocial interventions**


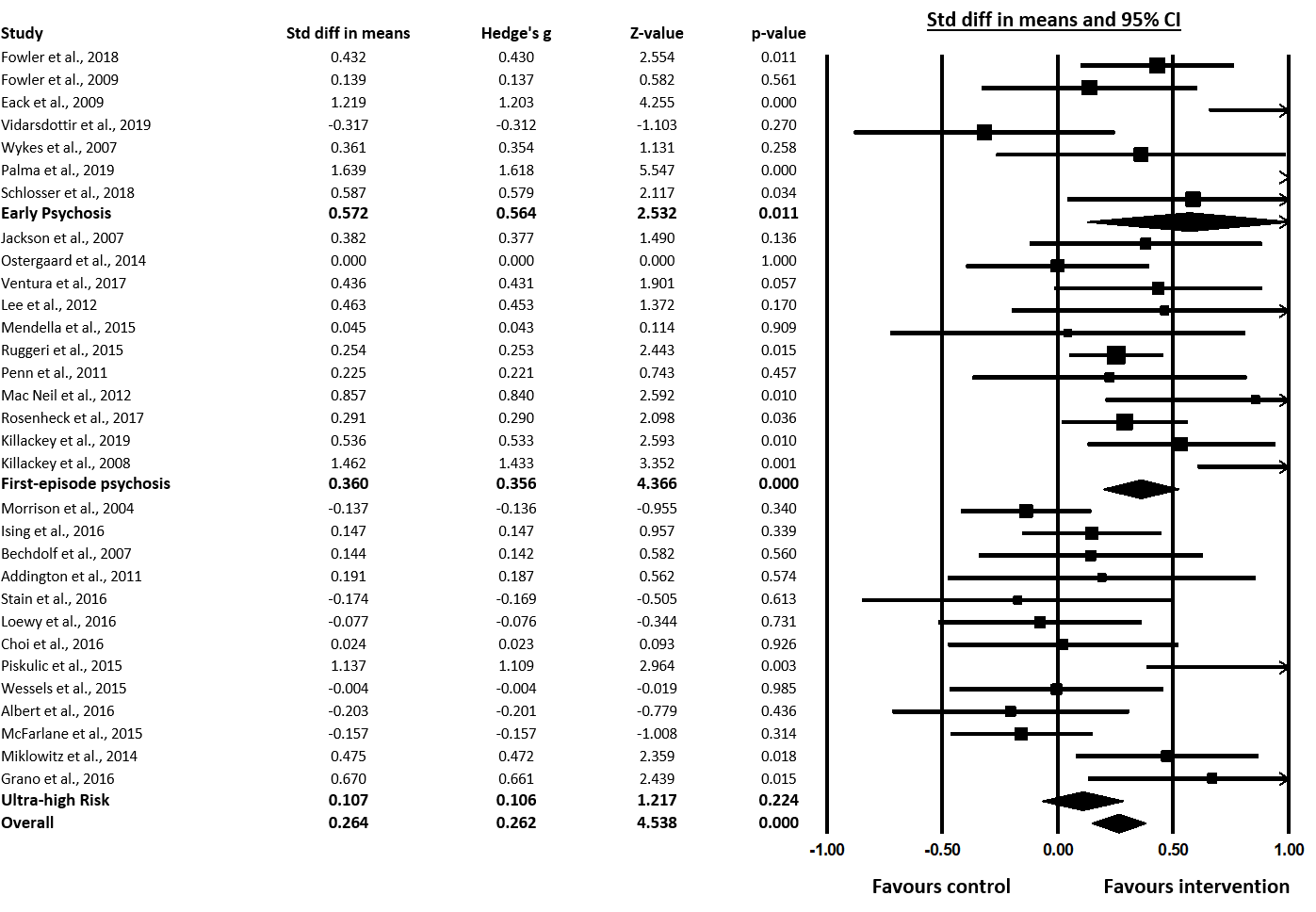


**Figure 5. Forest plot of overall subgroup analysis grouped by diagnosis.**


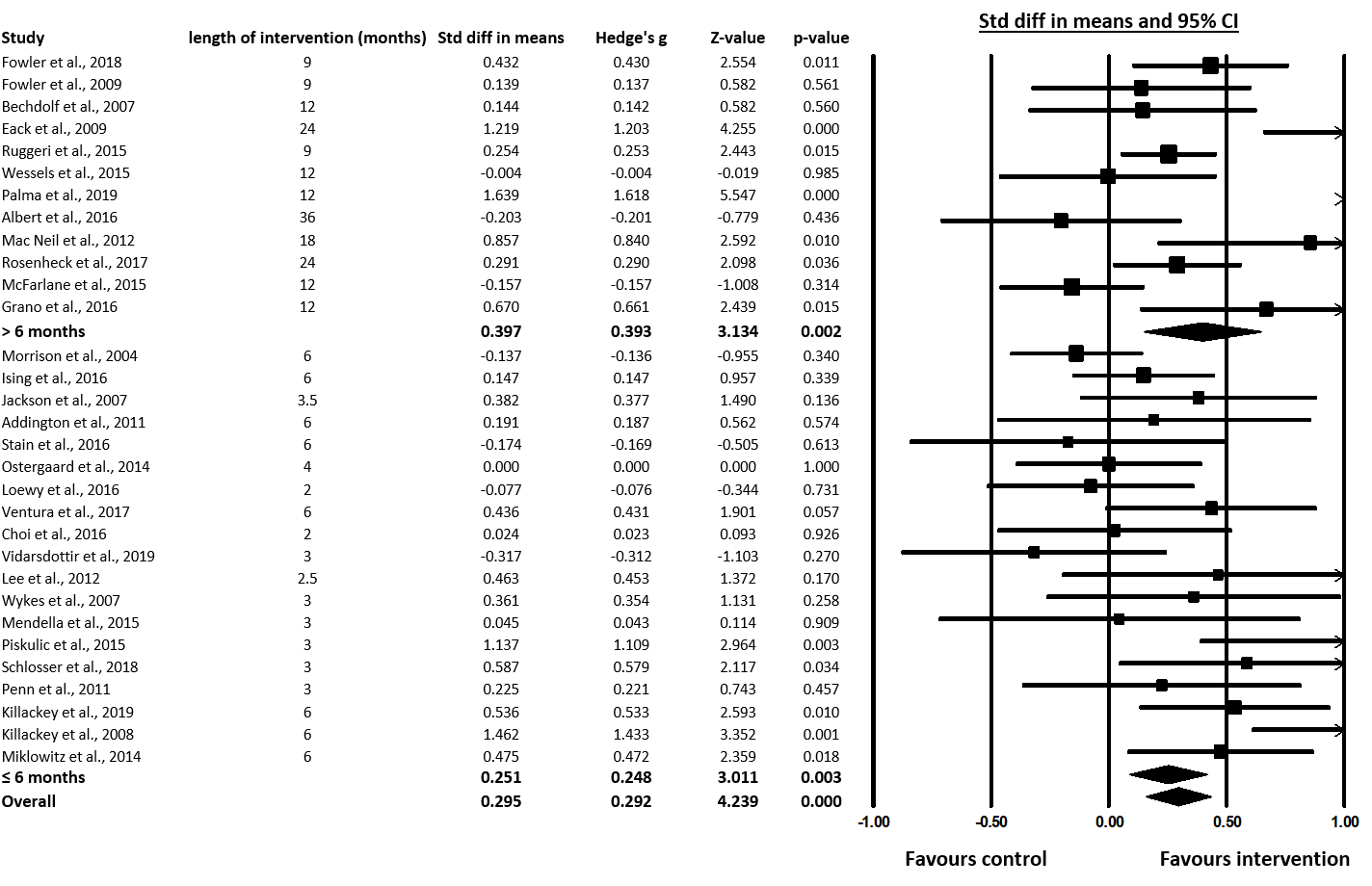


**Figure 6. Forest plot of overall subgroup analysis grouped by length of intervention in months.**


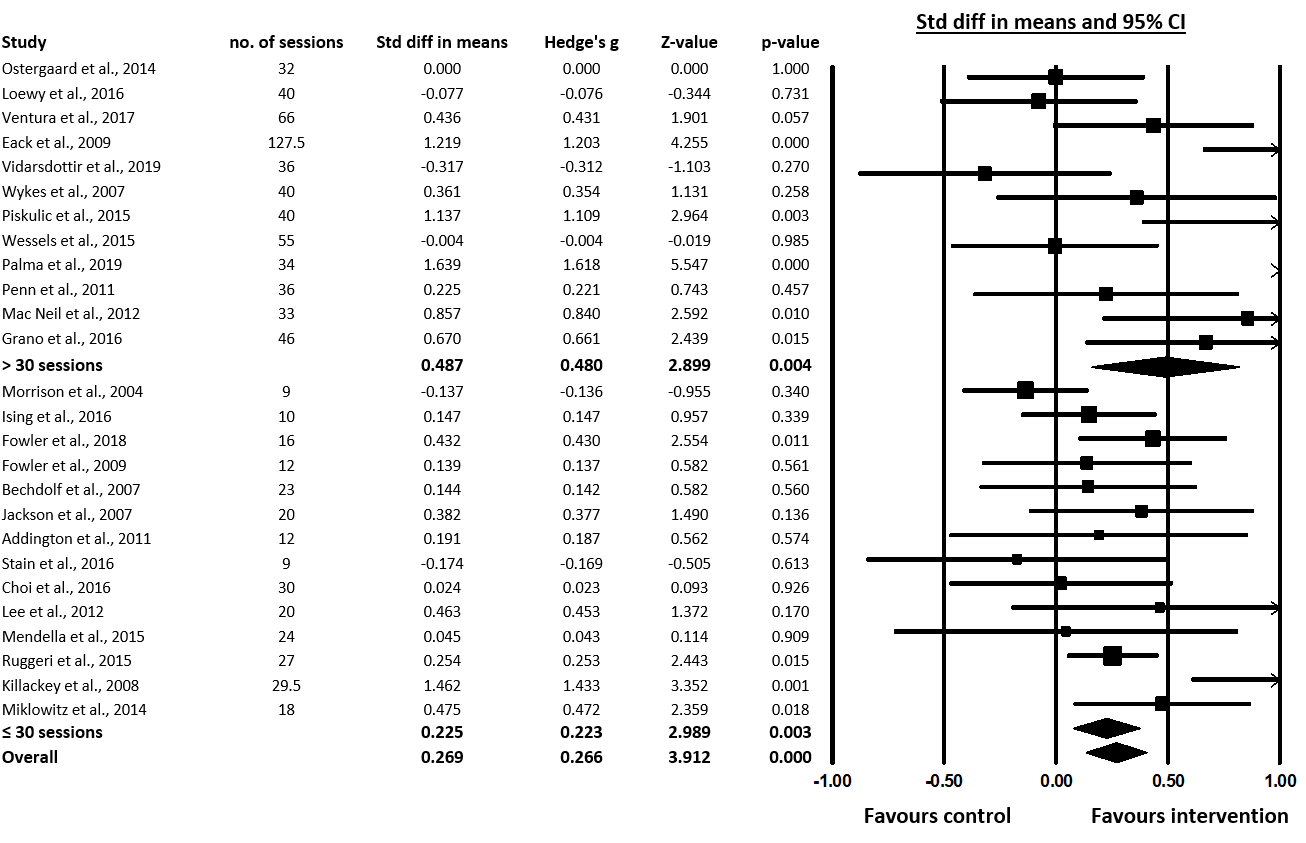


**Figure 7. Forest plot of overall subgroup analysis grouped by number of sessions.**


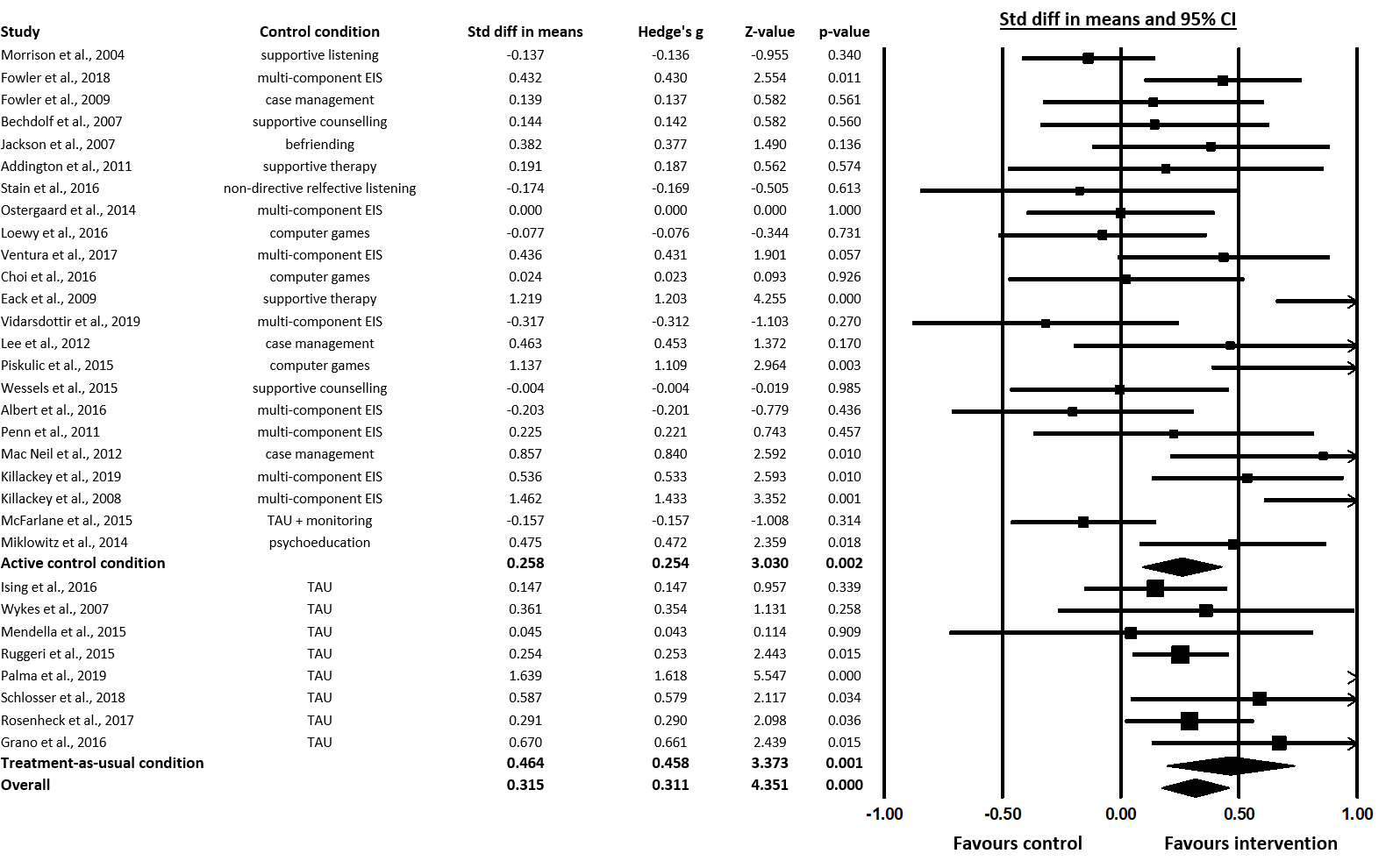


**Figure 8. Forest plot of overall subgroup analysis grouped by control condition.**


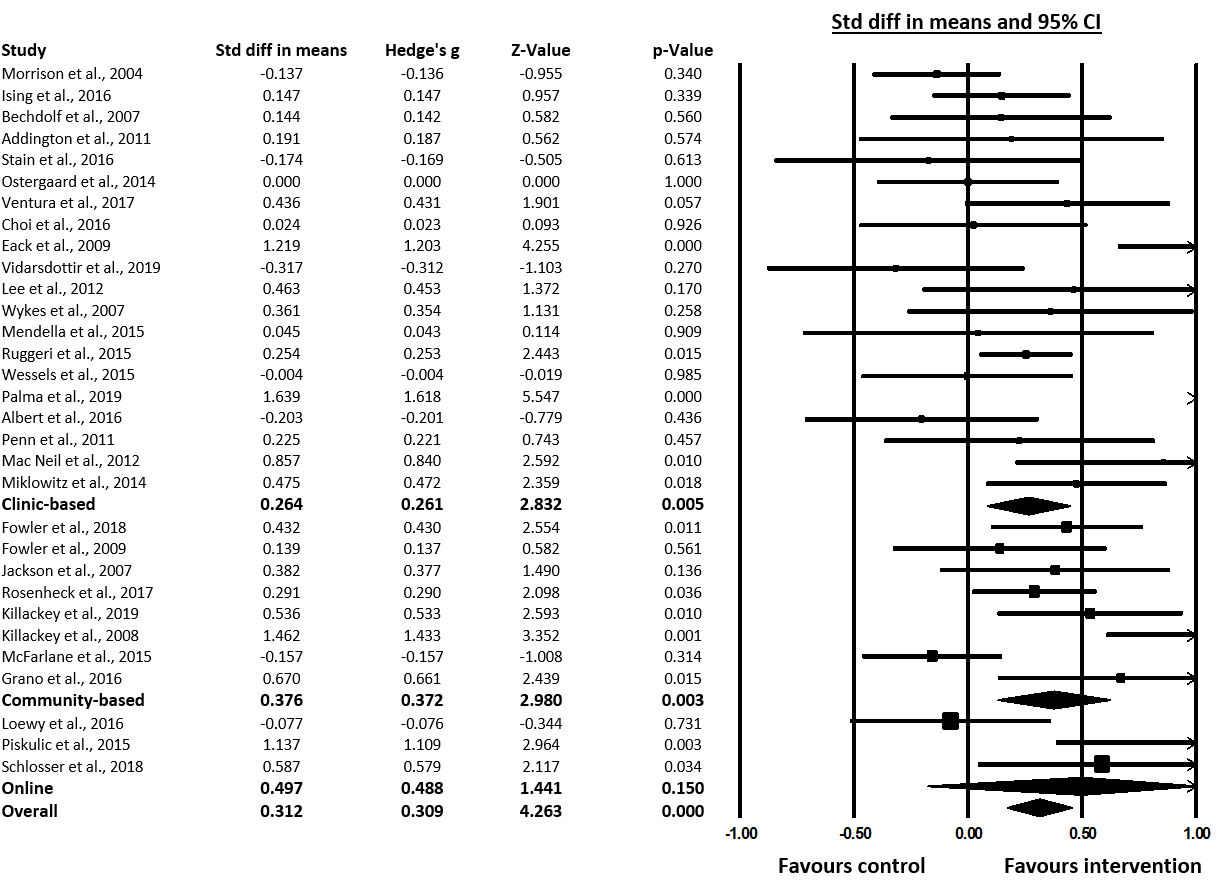


**Figure 9. Forest plot of overall subgroup analysis grouped by mode of delivery (clinic-based vs community-based vs online).**


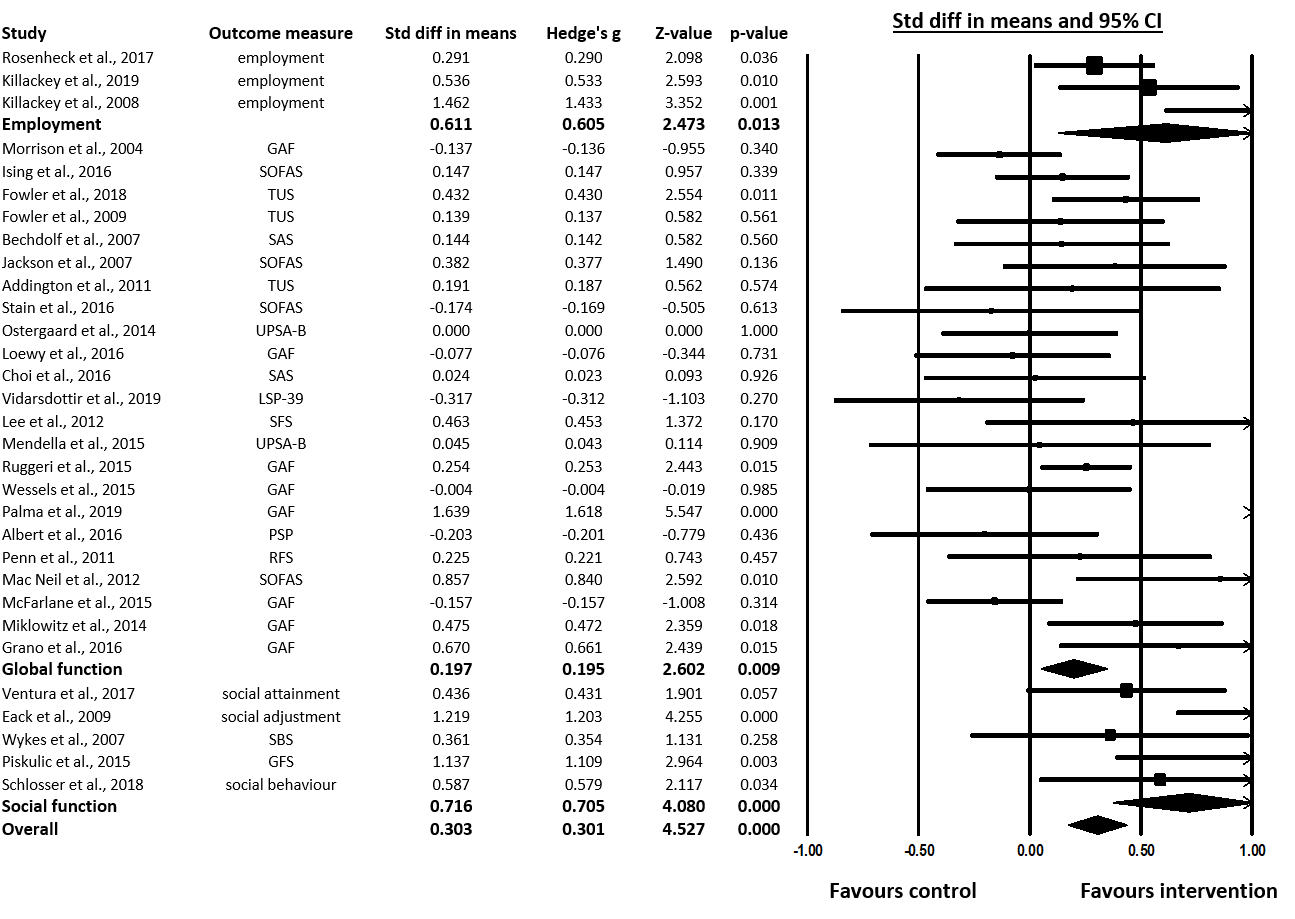


**Figure 10. Forest plot of overall subgroup analysis grouped by outcome measure (employment vs global function vs social function).**


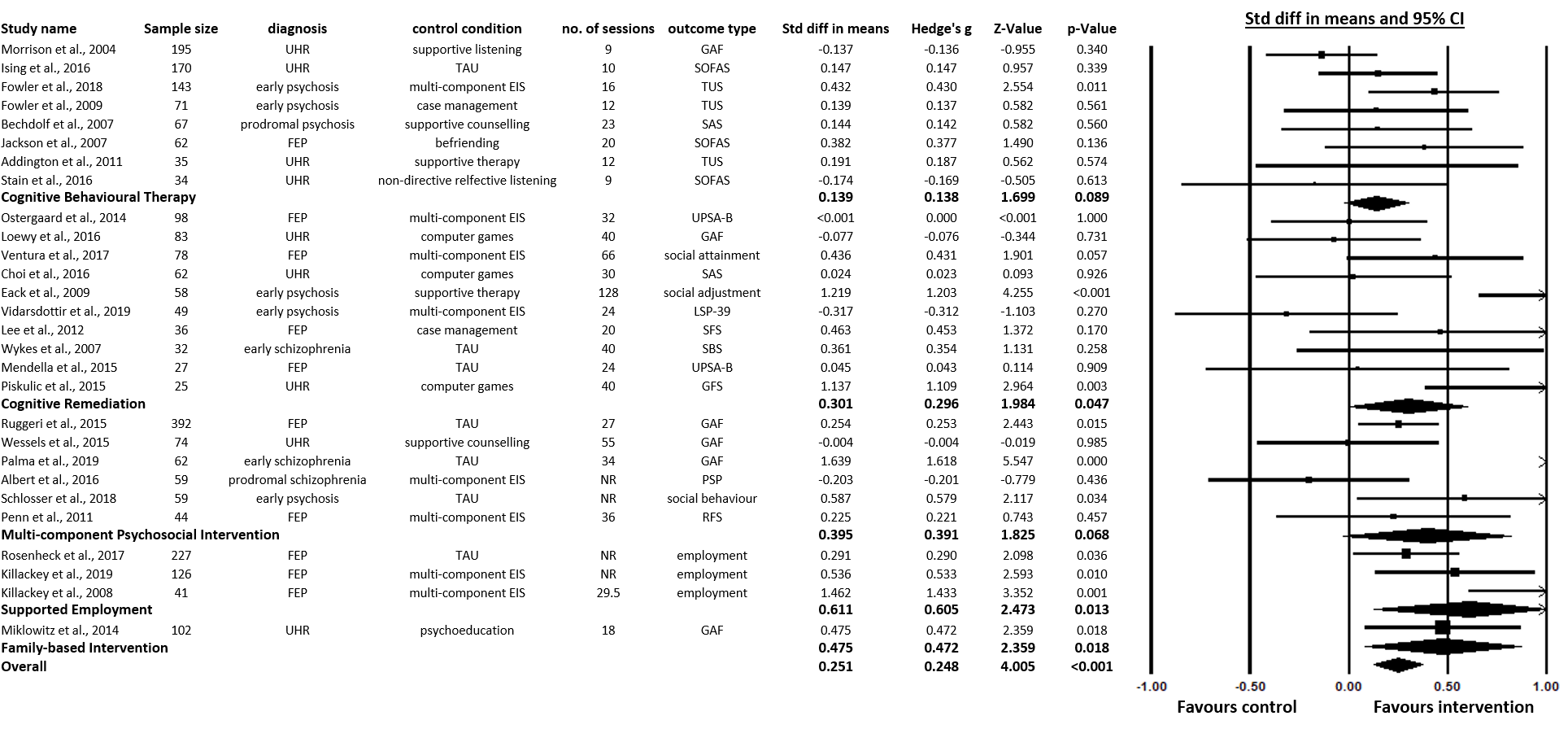


**Figure 11. Forest plot of summary statistics (SMD – Cohen’s d) for intervention groups and overall summary statistics for psychosocial interventions excluding non-RCT studies.**


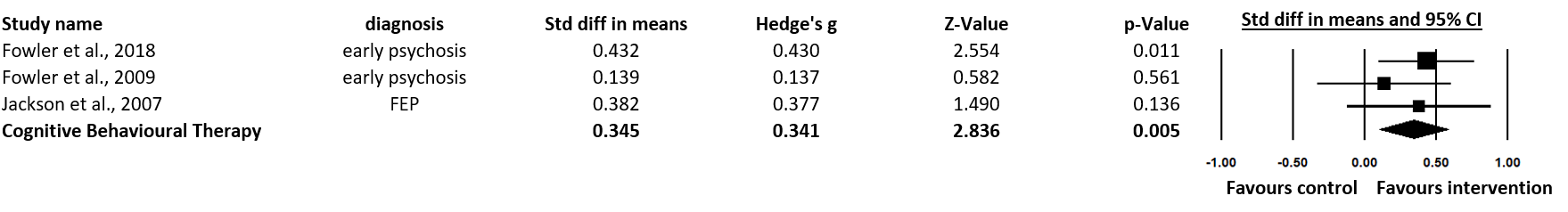


**Figure 12. Forest plot of summary statistics (SMD – Cohen’s d) for CBT excluding UHR studies.**
